# High performance, single crystal gold bowtie nanoantennas fabricated via epitaxial electroless deposition

**DOI:** 10.1038/s41598-023-38154-1

**Published:** 2023-08-07

**Authors:** Sasan V. Grayli, Saeid Kamal, Gary W. Leach

**Affiliations:** 1 Laboratory for Advanced Spectroscopy and Imaging Research, Simon Fraser University, 8888 University Dr, Burnaby, BC V5A 1S6 Canada; 2 Laboratory for Advanced Spectroscopy and Imaging Research, and 4D LABS, Department of Chemistry, Simon Fraser University, 8888 University Dr, Burnaby, BC V5A 1S6 Canada; 3 Present Address: Institute for Quantum Computing, University of Waterloo, 200 University Ave W., Waterloo, ON N2L 3G1 Canada

**Keywords:** Nanoscience and technology, Nanoscale devices, Nanophotonics and plasmonics, Materials science, Materials for optics, Nanophotonics and plasmonics, Optics and photonics, Optical materials and structures, Nanocavities, Electrochemistry, Chemistry, Physical chemistry, Optical spectroscopy, Raman spectroscopy

## Abstract

Material quality plays a critical role in the performance of nanometer-scale plasmonic structures and represents a significant hurdle to large-scale device integration. Progress has been hindered by the challenges of realizing scalable, high quality, ultrasmooth metal deposition strategies, and by the poor pattern transfer and device fabrication yields characteristic of most metal deposition approaches which yield polycrystalline metal structure. Here we highlight a novel and scalable electrochemical method to deposit ultrasmooth, single-crystal (100) gold and to fabricate a series of bowtie nanoantennas through subtractive nanopatterning. We investigate some of the less well-explored design and performance characteristics of these single-crystal nanoantennas in relation to their polycrystalline counterparts, including pattern transfer and device yield, polarization response, gap-field magnitude, and the ability to model accurately the antenna local field response. Our results underscore the performance advantages of single-crystal nanoscale plasmonic materials and provide insight into their use for large-scale manufacturing of plasmon-based devices. We anticipate that this approach will be broadly useful in applications where local near-fields can enhance light–matter interactions, including for the fabrication of optical sensors, photocatalytic structures, hot carrier-based devices, and nanostructured noble metal architectures targeting nano-attophysics.

## Introduction

The coupling of extended electromagnetic waves to planar metal/dielectric interfaces through surface plasmon polaritons (SPPs) or to nanometer-scale metal structures through locally resonant surface plasmons (LRSPs) leads to confined and amplified local fields that can be exploited for application in energy harvesting, sensing, spectroscopy, catalysis and imaging. The fate of these plasmonic excitations is intimately linked with the characteristics of the materials from which they are formed^1–6^. SPP propagation lengths, SP dephasing, decay, and decoupling are influenced strongly by material crystallinity and scattering processes that are induced by material defects, grain boundaries, and other material imperfections. Single-crystal plasmonic structures are expected to yield advantages over their polycrystalline analogues through reductions in optical absorption loss, grain boundary scattering and dissipation, while providing enhanced local fields derived from well-defined faceted nanostructures. In addition to these performance advantages, single-crystal plasmonics and nanophotonics will benefit from predictable and reproducible materials properties that lead to improved processing methods, production scale, device yields, and new applications, all of which are self-reinforcing and will help expand the scope and breadth of nanophotonic device applications.

While single-crystal materials have shown significant performance advantages in other applications^7–9^, single-crystal plasmonics has remained a challenge. Conventional deposition of plasmonic metals such as gold is typically carried out through physical vapor deposition (PVD) techniques and generally forms polycrystalline metal films and nanostructures. While deposition strategies and other protocols to mitigate the polycrystalline character of these films have been developed^10^, polycrystalline metal deposition can lead to compromised fabrication yields, as well as loss and dissipation that result in device inefficiency^11,12^, and remains a significant challenge in the field. We have recently developed an alternative approach to achieve ultrasmooth monocrystalline Au(100) films via electroless deposition from highly alkaline solutions of common gold salts onto Ag(100)/Si(100) substrates^13^ (Supplementary Information 1). The method is scalable to the wafer level, environmentally friendly, and represents a promising new approach to the integration of noble metal-based plasmonic structures into CMOS-compatible device architectures^14,15^. The high alkalinity environment of the electrolyte drives ligand replacement in the gold precursor AuCl_4_^¯^ (E° = 1.00 V) to form Au(OH)_4_^¯^ (E° = 0.57 V), circumventing galvanic replacement of the silver substrate (E° = 0.80 V), which would otherwise dominate at lower pH’s. Further, decreasing the rate of provision of electrons to the substrate (i.e. the rate of reducing agent oxidation) through the use of an unlikely reducing agent such as the hydroxide ion (4OH¯ → O_2_ + 2H_2_O + 4e¯ (E° = − 0.40 V)), limits the rate of metal complex reduction at the substrate surface, affording large area, uniform epitaxial noble metal deposition (Supplementary Information 2). Here, we use this approach to fabricate bowtie nanoantenna devices to provide a direct comparison between the performance characteristics of single-crystal and polycrystalline bowtie structures.

Bowtie nanoantenna devices are well-studied, simple dipole antenna structures comprised of two triangles separated by a small antenna feedpoint gap. The coupled nanostructures can provide intrinsically higher field enhancements than individual nanoparticles and the electromagnetic field in the gap of the antenna increases dramatically as the gap is decreased, generating gap fields that can be orders of magnitude larger than the incident electromagnetic field used to excite them. Plasmonically active bowties often provide some of the largest field enhancements and find application in surface enhanced spectroscopies, nonlinear optics, and nanophotonics^16,17^.

**Figure 1:**
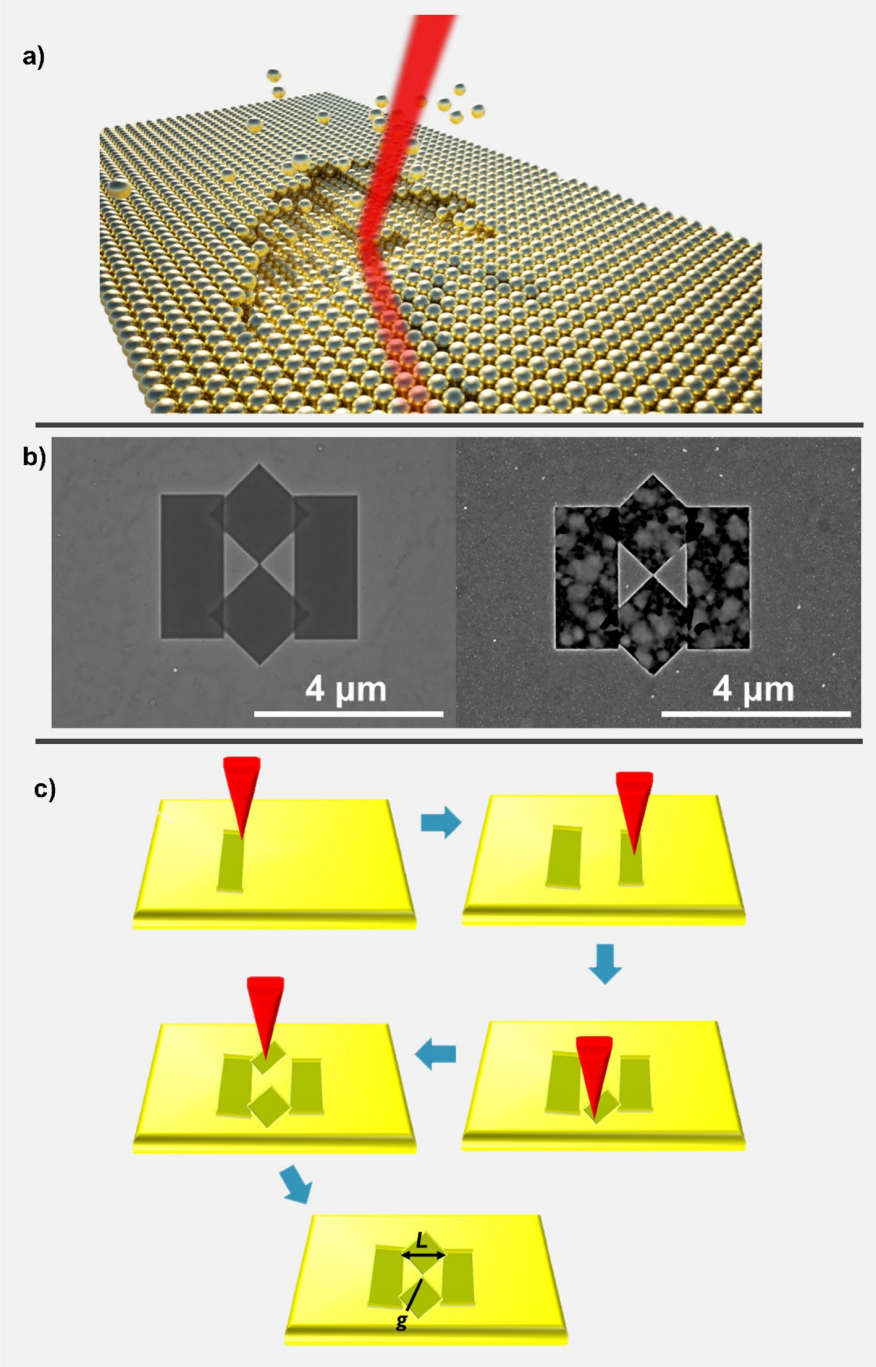
Bowtie nanoantennas fabricated on single-crystal and polycrystalline Au films. (**a**) Cartoon of the FIB milling of a single-crystal gold Au(100) film with an incident beam of Ga^3+^ ions (red) to realize a monocrystalline bowtie nanoantenna. (**b**) Top-view SEM images of bowtie antennas fabricated on monocrystalline (left) and polycrystalline (right) Au films, respectively. (**c**) Fabrication steps for the FIB milling of bowtie nanoantennas. For the structures fabricated here, L = 1560 nm, g = 20 nm.

Bowtie nanoantenna devices are fabricated using a Thermo Fisher Helios NanoLab 650 SEM/FIB system, using a focused gallium ion beam. Figure 1a illustrates the ion milling of material as the focused gallium ion beam is moved over surface regions in a serial fashion to create the bowtie nanoantennas on the surface. Our monocrystalline bowtie nanoantennas are fabricated in a 120 nm thick single-crystal Au(100) film deposited through epitaxial electroless deposition. Focused ion beam (FIB) milling of these single-crystal films results in high quality, low defect density, monocrystalline bowtie antenna structures (Fig. 1b (left)) (Fig. S1 left, Supplementary Information 3). We have also deposited 120 nm thick polycrystalline gold films by evaporation, utilizing a Si(100) wafer with a 5 nm Cr adhesion layer as a substrate, followed by patterning identical bowtie nanoantenna structures through FIB milling (Fig. 1b (right)). (Fig. S1 right, Supplementary Information 3).

The top-view scanning electron micrograph (SEM) of the milled single-crystal (left) and polycrystalline (right) bowtie structures (Figs. 1b and S1, Supplementary Information) reveal significant differences in the quality of patterning, with the milled regions of the monocrystalline film appearing highly uniform, and those of the polycrystalline film, much more irregular by contrast. The lack of milling uniformity in the polycrystalline films results from anisotropic, crystal direction-dependent ion milling rates and provides a bowtie structure defined by the remaining non-milled area, surrounded by a region of recessed roughened gold. Note that the pattern generation scheme involved milling rectangular and diamond regions sequentially (Fig. 1c). This process yields milled regions surrounding the bowtie that lie at different depths within the film which are separated by small vertical step edges. These regions can be seen readily (Fig. 1b, left) in the areas of overlap of the rectangular and diamond regions. The dimensions of the milled geometrical features were chosen to create a bowtie antenna with a length, L = 1560 nm, a gap, g = 20 nm, and height, h = 50 nm (Fig. S1, Supplementary Information 3).

### Dependence of yield and activity on film quality

Focused ion beam milling of $3 \times 10$ bowtie nanoantenna arrays was performed on single-crystal and polycrystalline gold films. The performance of the bowtie arrays was assessed by a Zeiss LSM 510 MP laser scanning microscope equipped with an LD Plan-Neofluar 63×/0.75 NA Korr objective lens, and a wavelength-tunable, ultrafast oscillator (Coherent Chameleon Ultra, 75 MHz repetition rate, 140 fs pulse duration) used to activate the antennas. Excitation of the bowtie nanoantennas at 780 nm leads to two-photon photoluminescence (2PPL) that is well-correlated with the locally resonant surface plasmon excitation of the structures. 2PPL imaging has been used extensively to characterize the resonant behaviour of plasmonic nanostructures^1,18–25^ and is utilized here as a measure of the nanoantenna plasmonic response and local field enhancement. In a previous study of bowtie nanoantennas fabricated from FIB milling of chemically grown single-crystalline gold flakes, Hecht and co-workers observed significant enhancement in 2PPL intensity from single-crystal antennas compared to polycrystalline structures^1^. These structures provide a stringent test of fabrication precision and yield, with the goal of uniform, reproducible and intense field confinement at the antenna feed points.

**Figure 2:**
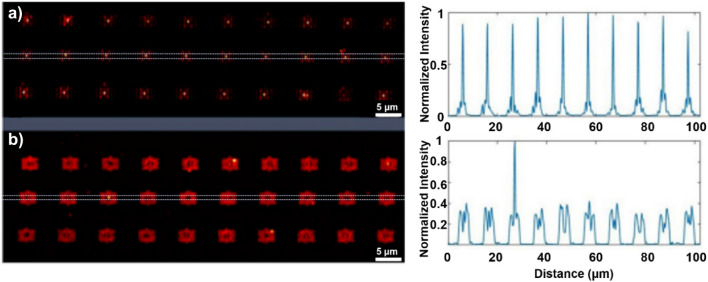
Yield and functionality of bowtie nanoantenna arrays. Two-photon photoluminescence (2PPL) intensity maps (left) and 2PPL cross-sectional gap intensity from the dashed regions of the centre row of antennas (right) fabricated from (**a**) single-crystal Au, and (**b**) polycrystalline Au films.

2PPL intensity maps of the bowtie arrays induced by 780 nm laser excitation are presented in Fig. 2 and highlight the primary performance differences between the mono- and polycrystalline nanoantennas. The 2PPL maps demonstrate that fabrication yield is greatly impacted by the material quality and by the resulting pattern transfer characteristics. The yield of monocrystalline bowtie antennas is close to 100% as measured by the appearance of an enhanced confined local near-field demonstrated in the 2PPL intensity at the 20 nm wide antenna feed points, and the relative uniformity of this 2PPL intensity for a majority of antennas, (Fig. 2a,b).

The identically milled structures in the polycrystalline gold film (Fig. 2b), show poor fabrication yield with only one device showing appreciable near-field intensity enhancement at its antenna feed point (middle row, bowtie 3 from the left), and little uniformity in 2PPL intensity from the others. Note that the fabrication differences between the mono- and polycrystalline structures (e.g. the presence of a Cr adhesion layer in the case of the polycrystalline antennas) can potentially lead to differences in the resonant response characteristics of the antennas. However, scanning of the laser wavelength in the vicinity of the expected resonant excitation spectra (780 nm) did not yield improvements in the emission characteristics of the polycrystalline antennas.

2PPL emission from the polycrystalline antennas (Fig. 2b) shows poor fabrication yield with few antenna structures displaying 2PPL gap intensity. While the integrated emission intensity from the polycrystalline antennas appears brighter than that from single-crystal devices, the vast majority of the 2PPL emission from polycrystalline devices emanates from the roughened recessed regions surrounding the bowties, and not from the antenna’s feed points, as desired (Fig. 2b, right). This “background” emission results from the roughened nature of the surrounding regions, as SP’s scatter from polycrystalline grain boundaries and material defects that arise from non-uniform and anisotropic milling. Further, the bright, localized 2PPL emission from monocrystalline antenna feed points (Fig. 2a, right) is significantly more intense than the average level of background emission emanating from polycrystalline devices, reflecting larger and more uniform field enhancement factors in the single-crystal bowtie gaps. Note that the observed 2PPL intensity from the antenna feed point regions is not a good measure of the gap field, since this luminescence results from two-photon excitation of the gold at the tips of the bowtie structures themselves, and there is no material capable of generating 2PPL in the bowtie gap regions, where the local plasmonic field is expected to be maximized. Aspects of the enhanced gap fields from the single-crystal bowties is further addressed below.

### Polarization dependence of the nanoantennas

**Figure 3:**
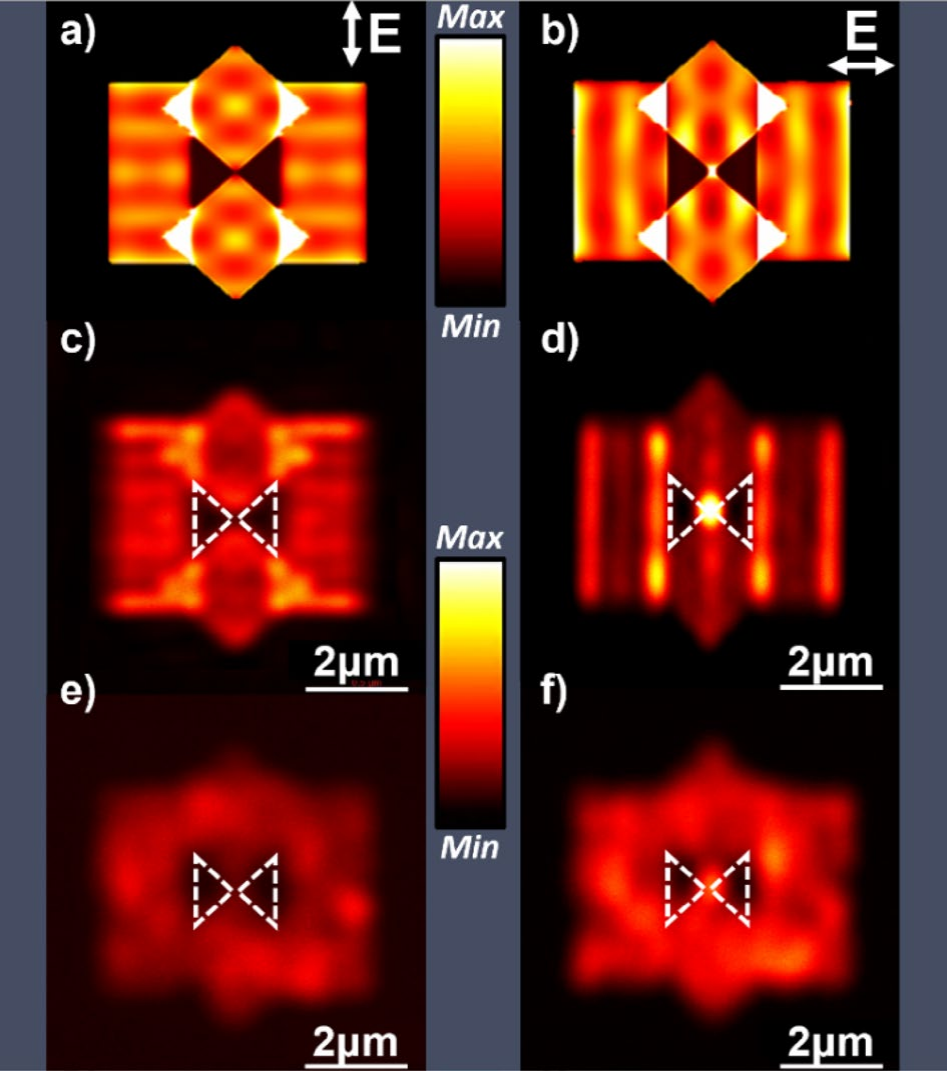
The effect of polarization on the activity of bowtie nanoantennas. FDTD modeled antenna response for (**a**) vertically and (**b**) horizontally polarized 780 nm excitation. Two photon photoluminescence intensity maps of a single-crystal bowtie nanoantenna (**c**) and (**d**) and polycrystalline bowtie nanoantenna (**e**) and (**f**) for vertically and horizontally polarized 780 nm excitation, respectively. 2PPL image intensities are normalized to the maximum intensity of each map.

The activity of the bowtie structures is known to be highly sensitive to electric field polarization. The bowtie nanoantennas fabricated on mono- and polycrystalline Au films were studied under vertically- and horizontally-polarized 780 nm laser irradiation at normal incidence. Their polarization-dependent 2PPL emission characteristics are illustrated in Fig. 3, along with a numerical calculation of the anticipated response of the bowtie nanoantennas, using finite-difference time-domain (FDTD) Ansys Lumerical (Supplementary Information 4). To compare the modelled and the experimentally measured antenna response accurately, the geometrical shapes employed in the FIB milling protocol of the fabricated devices were used to design the nanoantennas for the FDTD model. The single-crystal bowties are milled from 120 nm thick solution-deposited Au(100) onto Ag(100)/Si(100) substrates. Since silver is itself plasmonic, one may anticipate a contribution to the optical response from this underlayer. However, the gold thickness was chosen so that post milling, the ~ 75 nm Au(100) thickness corresponds to approximately three optical skin depths at the wavelengths employed in these experiments, resulting in little to no contribution from the underlying silver layer. This is supported by FDTD simulation of Au bowties modeled on a silver underlayer (as described and fabricated here), and those with a thick gold underlayer and no silver, which show only very minor differences in field distribution (Fig. S2, Supplementary Information 4).

The results in Fig. 3a,b represent the simulated device response for plane wave excitation at 780 nm for vertically and horizontally polarized incident light with respect to the bowties. As anticipated, the electric field distribution across the device is polarization-sensitive and shows field maxima lines that lie orthogonal to the polarization direction. The milling protocol results in the formation of recessed regions of the film that define local plasmonic cavities characterized by sharply edged walls. Light that is orthogonally polarized to the wall edges is edge-coupled into these cavities which are capable of supporting SP modes that appear as field intensity maxima in the FDTD simulations. These are readily visible as horizontal intensity maxima in the outer rectangular milled regions of the antenna under vertically polarized excitation (Fig. 3a), and as vertical intensity maxima in the horizontally polarized excitation (Fig. 3b). The mode patterns observed for the simulated milled structures in Fig. 3a,b in the immediate vicinity of the bowtie are further complicated by the plasmonic cavities defined by the diamond-shaped milled regions, leading to interference between modes and more complex intensity structure. Note that excitation of the structures with vertically polarized incident radiation that is orthogonal to the bowtie axis (Fig. 3a) results in no gap field at the antenna feed point while horizontally polarized incident radiation results in a confined local field in the bowtie gap, as expected.

Comparison of the plasmonic response of the modelled bowties with the fabricated bowties reinforces the significant differences in pattern transfer quality of the mono- and polycrystalline devices. Figure 3c,d display the corresponding 2PPL emission from a single-crystal bowtie under vertically- and horizontally polarized 780 nm short pulse excitation. Comparison of the FDTD modelled device response (Fig. 3a,b) and the experimentally observed single-crystal 2PPL characteristics (Fig. 3c,d) shows very good agreement. The experimental response displays horizontal intensity maxima upon excitation with vertically polarized light, and vertical intensity maxima upon horizontally polarized excitation, like those of the FDTD simulations. Figure 3b,d also show localized field maxima at the antenna feed point and where the milled diamond regions overlap with the milled rectangular regions and are readily apparent in both the simulated and fabricated structures. Note that differences between the 780 nm simulated and measured antenna responses may reflect differences between the plane wave excitation used for the FDTD simulation in comparison to the experimental measurement that employs a nominally gaussian ultrafast laser pulse of ~ 10 nm bandwidth, centered at 780 nm, focused on the sample with a high numerical aperture objective. Finite quality factors of the milled cavities will couple a range of incident wavelengths into the structures that can lead to SP mode interferences. Complex constructive and destructive interferences resulting from the multiple SP cavities that define the milled structure may contribute to intensity differences between the simulated and 2PPL intensity maps.

Comparison of the 2PPL emission response from polycrystalline bowties (Fig. 3e,f) shows very modest polarization dependence, the nature of which is significantly different from that observed from the monocrystalline antennas. Poor pattern transfer quality in the polycrystalline antennas leads to little or no well-defined mode structure as observed in the case of the single-crystal antennas. Since the polycrystalline bowties possess a rough surface morphology due to the anisotropic milling rates of the granular material from which they are fabricated, the rough surface morphology acts as a region made up of many nanostructures. Plasmonic excitation and rapid decay of these nanostructures through grain boundary and defect induced plasmon dissipation leads to 2PPL emission from the entire milled region with poorly defined polarization character. However, it should be noted that the overall intensity of 2PPL emission appears to be more intense for horizontally polarized excitation, presumably due to the enhanced coupling of light that is enabled by the bowtie antenna for this polarization. Further refinements in film quality, pattern transfer, and simulation accuracy are expected to improve the level of agreement between simulated and fabricated structures. Nevertheless, the high quality of material deposition enabled through our electroless deposition process, provides excellent agreement between simulation and 2PPL emission from the single-crystal bowtie structures.

### Plasmonic activity and field enhancement

Surface enhanced Raman spectroscopy (SERS) is a well-known and well-studied process in which the local excitation of SPs leads to a significant enhancement in the incoming and Raman-scattered light collected from surface molecules^26–29^. The locally excited electric field and the Raman enhancement can be achieved using nanoparticles and nanostructures made from plasmonic noble metals^27,28,30^, or with the help of nano-scaled devices with resonating cavities that can confine the excited SPs within very small gaps^31–39^. Here, the SERS response from the common Raman reporter molecule benzoic acid (BA) is used to compare the SERS efficiency as a measure of the relative magnitude of the field confinement for mono- and polycrystalline bowtie nanoantennas. In a receiving antenna, the maximum power gain is directly related to the maximum effective area of the antenna, A_e_, which is calculated through:

1 $${\text{A}}_{{\rm e}}=\frac{{\lambda}^{2}}{{4}{\pi}}$$

where *λ* is the wavelength of the incident photon^40^. Since the bowtie nanoantennas on mono- and polycrystalline Au films are identical in design, we expect the *A*_*e*_ to remain constant across the fabricated devices on both surfaces and therefore the difference in the performance can be attributed to the quality of the materials. The field confinement magnitude at the gap of the plasmonic bowtie nanoantennas is linked to the coupling efficiency of photons to SPs, which in turn, is a function of surface quality of the film from which the device is made^1,4–6^. The surface roughness of the polycrystalline devices negatively impacts the intensity of excited SPs at the bowtie feed point by enabling photon-SP decoupling at grain boundaries and material defects, thereby reducing the magnitude of the field at the gap. This route for SP intensity decay is minimized for the monocrystalline Au nanoantennas, resulting in a larger gap field.

**Figure 4:**
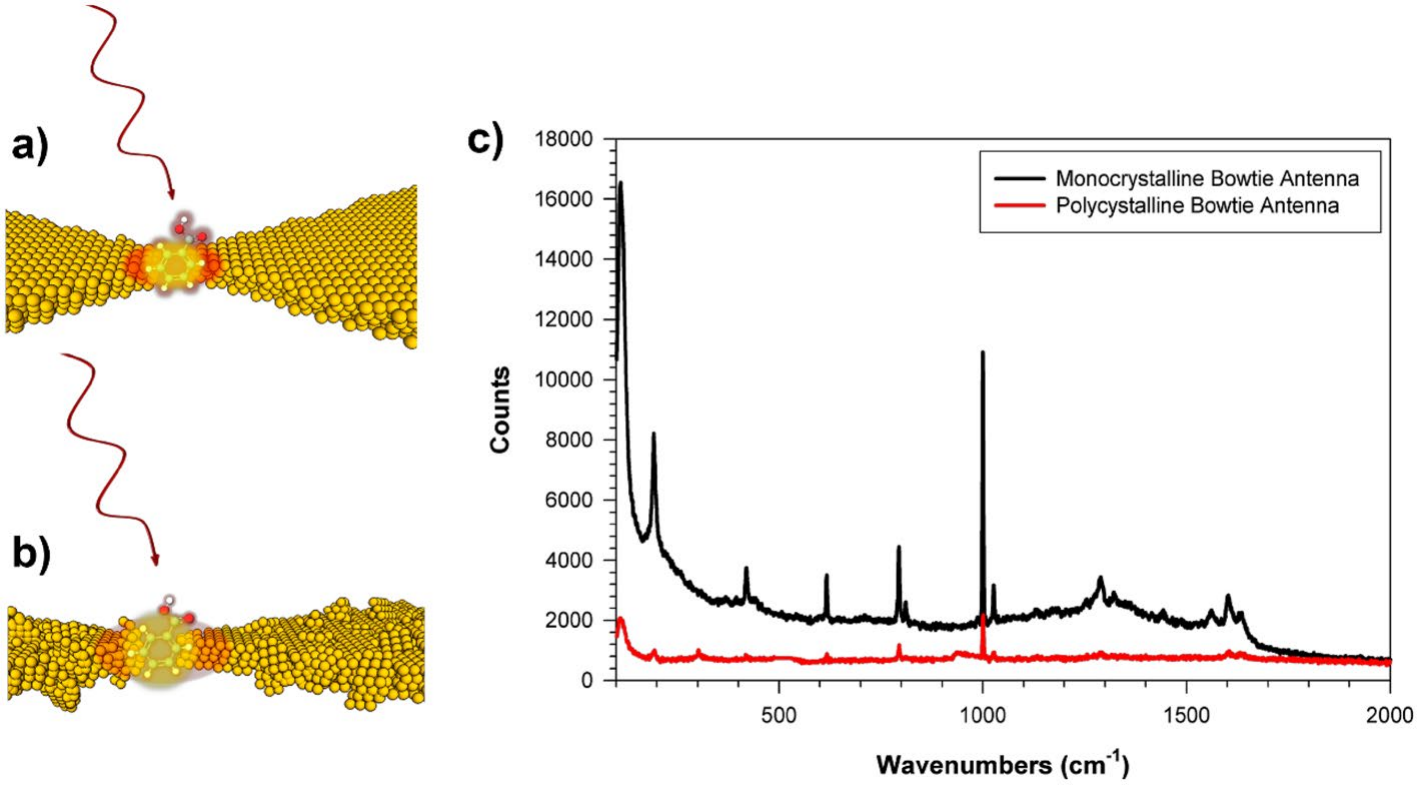
Surface enhanced Raman scattering (SERS) of benzoic acid (BA). In (**a**) a monocrystalline Au bowtie nanoantenna and (**b**) a polycrystalline Au bowtie nanoantenna are depicted. (**c**) The SERS spectra of BA from single-crystal (black) and polycrystalline (red) bowtie nanoantennas obtained with 785 nm excitation, illustrating enhanced SERS activity from single-crystal bowties. See text for vibrational assignments.

Both mono- and polycrystalline devices were coated with 10 μL of 0.02 M BA in methanol, by drop casting, followed by solvent evaporation. SERS spectra were collected using a Raman microscope/spectrometer (Renishaw Invia) equipped with a fiber-coupled continuous wave 785 nm diode laser, as the excitation source and a 50 × objective. The Raman spectra were acquired at 50% incident laser intensity, using 10 s CCD exposure times. The bowties were separated far enough apart from one another such that under these illumination conditions, Raman data from single devices could readily be acquired. The SERS spectra from BA-coated bowties appear in Fig. 4c and are representative of the mono- (Fig. 4a) and polycrystalline (Fig. 4b) responses from many bowtie measurements. The observed spectral response of both single-crystal and polycrystalline bowties is consistent with the observed SERS response from previous BA studies^41–48^, indicating that with this deposition protocol we probe BA multilayer films. We observe weak and broadened SERS spectroscopic signatures consistent with BA interacting with the gold substrate through gold-carboxylate interactions (ν_s_ COO^–^, 1375 cm^−1^; ν_as_ COO^–^, 1570 cm^−1^)^44,45^ visible in the enhanced single-crystal bowtie spectrum. However, the dominant spectral response from both single-crystal and polycrystalline bowties is from multilayer BA, dominated by BA in its dimeric form, which is stabilized by strong intermolecular hydrogen bonding forces and gives rise to relatively narrow and intense Raman features from its fundamental skeletal ring distortion vibrations (ν_12_ 1000 cm^−1^; ν_18a_ 1028 cm^−1^; ν_11_ 798 cm^−1^; ν_10a_ 816 cm^−1^; ν_6a_ 424 cm^−1^; ν_6b_ 620 cm^−1^; ν_8a_ 1610 cm^−1^), where we have used the mode numbering convention of Varsanyi^41^. The spectra of multilayer BA also display activity due to the carboxylic acid functional group in the 1290 cm^−1^ region (ν C–OH), and at 1630 cm^−1^ (ν C=O). We also observe intense low frequency features that are well-correlated with the dimer structure (C-phenyl out-of-plane bend 190 cm^−1^; inter-monomer stretch 120 cm^−1^), in agreement with the low frequency modes of BA dimers measured via DFT calculation, inelastic neutron scattering^46,47^, and infrared spectroscopy^48^.

The SERS data suggests that the larger enhancement from single-crystal antennas can be attributed to the quality of the Au film from which the devices were fabricated. Evidently, the single-crystal nanostructures support larger near-field gap intensities than their polycrystalline counterparts, suggesting significant advantages in the use of single-crystal plasmonic materials. Performance improvement and antenna efficiency are expected to result in part from device material conductivity^49,50^, reducing conduction losses as the captured field of incident photons is directed toward the antenna feed point. At optical frequencies, ohmic losses occur in close proximity to the surface and therefore material quality and conductivity of the metal plays a critical role in determining device impedance^40,49^. We have previously reported the improved conductivity of the solution-deposited monocrystalline gold films compared to their vapor-deposited counterparts^13^. Four-point probe measurements on both mono- and polycrystalline gold surfaces indicate that the conductivity of the single-crystal gold surfaces are greater by approximately a factor of 20 over the polycrystalline films. While the drop-cast deposition method suffers from potential concentration variations, systematically larger SERS response from single-crystal bowties suggest that their enhanced response is material-related, and likely originates from enhanced local gap fields. Method development to quantify the gap field more directly without the uncertainties of inhomogeneous analyte concentrations in the plasmonically enhanced bowtie gap regions are currently underway in our laboratory.

## Conclusions

We have demonstrated a new scalable and green solution-deposition method for the fabrication of large area single-crystal Au(100) films. The subtractive manufactured single-crystal plasmonic bowtie nanoantennas with improved fabrication and performance yields, was compared to their polycrystalline counterparts. We have presented a direct and quantitative comparison of the performance of mono- versus polycrystalline plasmonic bowtie nanoantennas. Single-crystal bowties were fabricated via FIB milling of Au(100) films deposited by epitaxial electroless deposition from highly alkaline deposition baths onto Ag(100)/Si(100) substrates. Polycrystalline antennas were fabricated through an identical patterning protocol on polycrystalline films deposited by Au evaporation onto a Si(100) wafer containing a 5 nm thick Cr adhesion layer. The quality and yield of pattern transfer onto single-crystal films far surpasses those of polycrystalline films and leads to significant performance advantages of the single-crystal devices. These include improved device uniformity, enhanced intensity of local near-field distributions, and the ability to model these distributions accurately. Our single-crystal bowties show greater SERS enhancement factors than their polycrystalline counterparts through reduced photon-surface plasmon decoupling and absorption loss, providing greater local gap fields that enhance light-matter interactions. While this study has focused specifically on bowtie nanoantennas, we anticipate that the design and performance of other plasmonic structures will also benefit from single-crystal materials through their superior thermal, mechanical, and optical properties, giving rise to predictable and reproducible material behaviors and processing improvements that will help to expand the scope of their nanophotonics applications.

### Supplementary Information

Supplementary Information.

## Data Availability

The data that support the plots and other findings of this study are available from the corresponding author upon reasonable request at gleach@sfu.ca.
