# Supplementary material for: High performance, single crystal gold bowtie nanoantennas fabricated via epitaxial electroless deposition
Source: Sci Rep. 2023 Aug 7;13:12745. doi: 10.1038/s41598-023-38154-1 (PMC10406868; doi:10.1038/s41598-023-38154-1)
Supplement: Supplementary file 1 — Supplementary Information. [file 41598_2023_38154_MOESM1_ESM.pdf]

Supplementary Information

High-performance, single-crystal gold bowtie nanoantennas fabricated via epitaxial electroless deposition

Sasan V. Grayli[1], Saeid Kamal[2], and Gary W. Leach[3,*]

[1] current address: University of Waterloo, Institute for Quantum Computing, 200 University Ave W., Waterloo, ON N2L 3G1 Canada, [2] Simon Fraser University, Laboratory for Advanced Spectrocopy and Imaging Research, 8888 University Dr, Burnaby BC V5A 1S6 Canada, [3] Simon Fraser University, Department of Chemistry, Laboratory for Advanced Spectrocopy and Imaging Research, and 4D LABS, 8888 University Dr, Burnaby BC V5A 1S6 Canada

1. Monocrystalline silver deposition on Si(100)

Silver Ag(100) deposition was carried out using a Kurt J. Lesker Company PVD-75 thermal evaporation tool with a base pressure of <2 × 10^{-7} Torr. Ag (99.99% Kurt J. Lesker Company) was evaporated from an alumina coated tungsten wire basket. The substrate was heated via a backside quartz lamp and the temperature was monitored with a K type thermocouple attached to the backside of the sample chuck assembly. Deposition was carried out at a substrate temperature of 340 ºC and a rate of 3 Å/s. Prior to Ag deposition, substrates were immersed in either dilute HF acid solutions (10:1 with de-ionized water), or similarly diluted commercial buffered oxide etch solutions (BOE, CMOS Grade, J.T. Baker Inc.), to remove the native oxide layer from the surface of the silicon wafer. All activities, prior to characterization of the films, were carried out under class 100 clean room conditions or better.

2. Electroless deposition of single-crystal gold on Ag(100) substrates

A 1 x 1 cm^2 Ag(100) substrate was used as the surface on which to grow a 120 nm thick monocrystalline Au(100) film through epitaxial electroless deposition. The Ag substrate was submerged in 10 mL of 1 M NaOH which acted as the deposition bath. Then 250 µL of 0.025 M of $HAuCl_4$ solution was added to the deposition bath (10 mL NaOH). The solution was placed in a water bath where its temperature was kept at 60°C for 60 minutes undisturbed to grow a ~120 nm thick monocrystalline Au(100) film on the Ag(100)/Si(100) substrate. The sample was then washed with distilled water and sonicated in isopropanol alcohol for 60 s and air dried.

3. Gold bowtie nano-antenna fabrication

An FEI Helios Focused-Ion beam (FIB) tool (4D LABS) was used to fabricate the gold bowtie nano-antennas. The process was carried under the pre-set conditions in the tool for Au films, in which the desired milling depth was 50 nm. The ion beam current was set at 7.7 pA for the 30 kV operating voltage. Under

these conditions, for 50 nm depth etching the dose was set to be 33 pC/μm^2 and this value was doubled for the milling the monocrystalline Au film. The exposure time for fabrication of bowtie nano-antennas on the monocrystalline Au film was also increased by a factor of 2 over the parameters used for milling polycrystalline films to achieve a milling depth of 50 nm, due to the lower material removal rate for single-crystal Au. Figure S1 shows the fabricated bowtie antenna on both monocrystalline and polycrystalline Au achieved under these etching conditions.

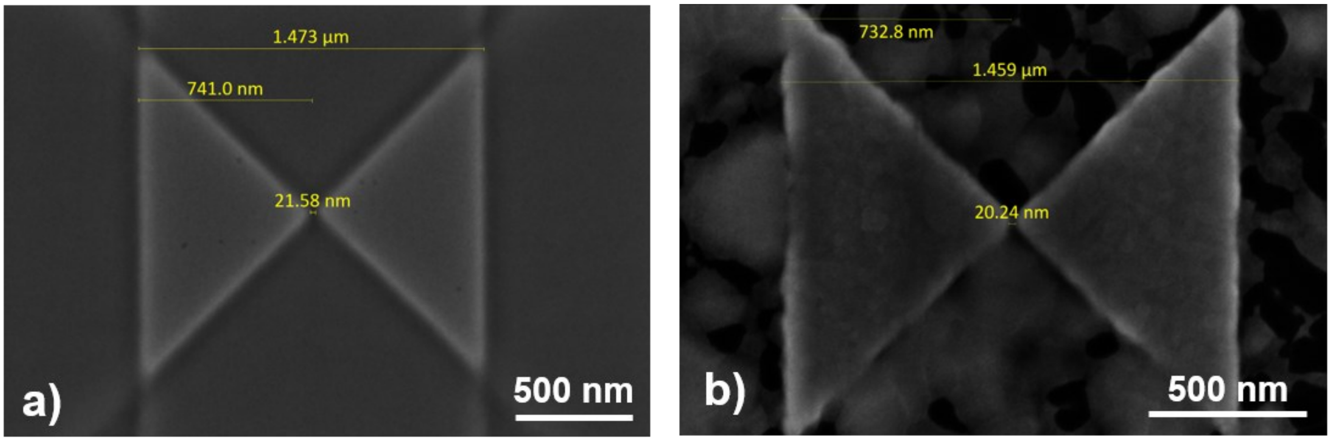

Fig. S1: The fabricated Au bowie nano-antennas on a) monocrystalline Au(100) and b) thermally evaporated polycrystalline Au.

4. Finite-difference time-domain simulations

The FDTD analysis was carried out using Lumerical Solutions FDTD tool to simulate the electric field distribution across the surface of the fabricated bowtie for comparison with the experimental results. The design of the structures input to the FDTD model were as close as possible to the fabricated structures for more accurate analysis. The images shown in the figure 3a) and b) of the manuscript, are from a power monitor placed 50 nm above the structure at 0° and 90° polarization respectively. The source used in this simulation was a plane wave with a bandwidth from 730 nm to 830 nm (centered at 780 nm). A uniform mesh with 1 nm x 1 nm x 1 nm size was used over the region under simulation with 1000 fs simulation time. The dimension of the FDTD simulation area was 5 x 5 x 2 μm^3 (3D simulation) and the mesh accuracy of the simulation was high with a 0.25 nm minimum mesh step. Perfect matching layer (PML) boundary conditions were employed with 12 PML layers in all directions and 0.0001 PML reflection. The substrate on which the bowtie nano-antenna was designed, was a 10 x 10 x 2 μm^3 cuboid and the optical constants employed for the simulation were those provided with the Lumerical software (Au (Gold)-CRC).

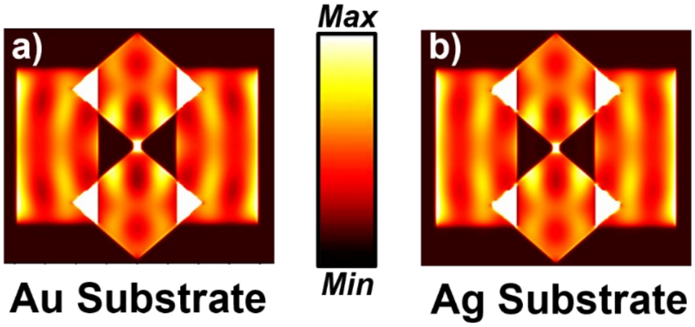

Fig. S2: FDTD simulation of a FIB-milled bowtie nanoantenna patterned on a thick Au substrate without silver (left) and the same structure patterned on a 120 nm thick Au film on a thick silver substrate.
